# Marbling score, cholesterol, and physical–chemical content of male Bali beef fed fermented pineapple peel

**DOI:** 10.5455/javar.2022.i610

**Published:** 2022-09-30

**Authors:** Bulkaini Bulkaini, Dahlanuddin Dahlanuddin, Tirta Ariana, Djoko Kisworo, Maskur Maskur, Mastur Mastur

**Affiliations:** 1 Faculty of Animal Science, University of Mataram, Mataram Lombok, Indonesia; 2 Faculty of Animal Husbandry, University of Udayana, Badung, Indonesia

**Keywords:** Bali cattle, fermentation, cholesterol, marbling

## Abstract

**Objective:**

The study was conducted to determine the marbling score, fat and meat color, cholesterol, high-density lipoprotein (HDL), low-density lipoprotein (LDL), and physical–chemical content of male Bali beef fed fermented pineapple peel.

**Materials and Methods:**

Twelve heads of male Bali cattle with an initial weight of 168.46 ± 11.95 kg were put into individual cages at random based on a completely randomized design with three treatments and four heads of Bali cattle as replicates, namely T0 = NG + (39% MC + 61% RB + 0% fermented pineapple peel); T1 = NG + (10% MC + 70% RB + 20% fermented pineapple peel with yeast culture); and T2 = NG + (15% MG + 65% RB + 20% pineapple peel fermented by lactic acid bacteria). The sample of Bali cattle meat used in testing the research variables was the LD muscle, with as many as 24 samples for each treatment. The data were analyzed based on the analysis of variance using the Statistical Product and Service Solutions software program, following Duncan’s test with 5% confidence.

**Results:**

The results showed that the treatment T2 could increase the marbling quality of the meat from 2.58% to 4.00%. The cholesterol content (80 mg/100 gm), HDL (60 mg/100 gm), LDL (10 mg/100 gm), water-holding capacity (36.10%), cooking loss (29.16%), tenderness/shear force (4.08 kg/cm^2^), crude protein (22.99%), crude fat (4.23%), and meat collagen (1.65%) were determined.

**Conclusions:**

Adding 20% of fermented pineapple peel by lactic acid bacteria to the ration can improve the quality of marbling, produce cholesterol, and the physical–chemical value of meat that meets the Indonesian National Standard.

## Introduction

Bali cattle (*Bos javanicus*) domesticated on the island of Bali are the primary native cattle breed in Indonesia. They adapt well to the Indonesian climatic and socioeconomic conditions, particularly the hot tropical environment, and can thrive on low-quality feeds [1]. More than 30% of the total domestic beef is produced by Balinese cattle [2].

Many experiments have been carried out on the feed management and breeding of Bali cattle, but these studies were mostly related to cattle productivity indicators such as characteristics of body weight, body size, breeding, feeding [3–6], and growth [7]. Specific experiments related to the marbling score and cholesterol content of Bali cattle beef are still limited. The cholesterol content of meat is a consumer consideration when consuming meat because it is associated with the risk of high cholesterol. The high or low quality of marbling and cholesterol content of meat depends on the quality of feed [2].

Bali cattle fed with concentrates have a higher marbling score than beef from Bali cattle fed a variety of grasses [8]. Bali cattle fed with fermented cocoa pods had a marbling score of 2.65% [9], while Bali cattle supplemented with commercial concentrate had a marbling score of 3.91% [2].

Cholesterol and triglyceride levels have recently received much attention because both cholesterol and triglycerides are factors considered to cause coronary heart disease [8]. The cholesterol content of meat depends on several factors, including cattle body weight, age, sex, and type of feed. The cholesterol content (mg/100 gm) of beef, goat meat, lamb, pork, buffalo meat, and venison is 73.1, 75, 66, 69, 62, and 67, respectively [7].

Physical and chemical properties of meat consist of pH, water-holding capacity (WHC), cooking loss, tenderness, protein, fat, and water content [8]. The physical and chemical properties of meat are influenced by pre-slaughter factors that include sex, age, body condition, quality of feed, pre-slaughter treatment, and after-slaughter factors that include the provision of meat tenderizing enzymes, electrical stimulation, low-temperature storage, and cooking methods [10].

Improving feeding management by paying attention to the quality and continuity of feed availability is one strategy to get beef with a high marbling score, low cholesterol content, and balanced physicochemical properties. The sources of noncompetitive local materials that have the potential to be used as ingredients for Bali cattle rations are pineapple peel waste, and pineapple peel waste production for 2021 in the West Nusa Tenggara region of Eastern Indonesia was recorded at 16.400 tons, with pineapple peel waste production of 3.936 tons/year [11].

The nutritional value and palatability of pineapple peel should be increased by fermentation using commercial yeast culture inoculums [12]. Fermentation of pineapple peel waste using yeast culture (*Saccharomyces cerevisiae*) at a level of 8%, a temperature of 27°C, and pH of 4 can produce an optimal volume of alcohol [13]. Using 2% yeast culture for 4 days in cocoa bean fermentation is the best treatment, producing 2.10% pectin content, 0.70% reducing sugar, and a pH of 3.9 [14]. Using fermented pineapple peel in broiler chicken rations at a level of 22.5% can reduce the cholesterol content of meat from 152.80 mg/100 gm to 144.00 mg/100 gm [15]. The addition of fermented pineapple peel in commercial broiler chicken feed reduced breast meat’s cholesterol level from 0.84 ± 0.14 mg/100 gm to 0.63 ± 0.12 mg/100 gm [16].

Male Bali cattle fed bio-plus fermented cocoa pod husks at 30%, 40%, and 50% in ration produced meat with a marbling score of 3.34%, 3.72%, and 3.53%, respectively, with the respective meat cholesterol content of 77.25 mg/100 gm, 66.38 mg/100 gm, and 66.50 mg/gm [17]. The physical–chemical value of male Bali beef, which is reared extensively in the pastures of Bima, West Nusa Tenggara at the age of 2.5–3 years of slaughter, has a meat tenderness value of 5.16 kg/cm^2^, cooking loss (44.95%), water holding capacity (21.01%), meat protein (18.16%), and 1.92% longissimus dorsi meat fat [10]. Based on the description above, a study was carried out to determine the marbling score, fat and meat color, cholesterol, high-density lipoprotein (HDL), low-density lipoprotein (LDL), and physical–chemical content of male Bali beef fed fermented pineapple peel.

## Materials And Methods

### Ethical approval

The male Bali cattle used in this experiment have been approved by the Ethical Committee of the Faculty of Animal Science, University of Mataram, Indonesia (approval number: 07/UN18.F2/EC/2021, March 05, 2021). The animals in this study have been handled professionally; during the study, male Bali cattle were placed in individual cages with a size of 1.5 x 2 m^2^. During the research, the animals were still given food and drink according to the treatment. At the end of the study, the experimental animals were slaughtered according to the rules of Islamic law at the slaughterhouse to get samples of the research meat.

### Materials

Twelve male Bali cattle with an initial weight of 168.46 ± 11.95 kg were used. The ration composed of native grasses and concentrate, which consisted of fermented pineapple peel with yeast culture, fermented pineapple peel with lactic acid bacteria, milled corn, rice bran, and molasses. The animals were raised in 1.5 × 2 m2 individual pens equipped with feed and drinking water.

### Pineapple peel fermentation

The study began with the pineapple peel fermentation process using the facultative anaerobic method [12]. Pineapple peel fermentation applies two types of commercial inoculums such as yeast culture (*S. cerevisiae*) and lactic acid bacteria. Fermentation of pineapple peel was carried out with the following procedure: (1) pineapple peel was cleaned, cut into 2 × 3 cm in size, dried under the sun, and then ground to become pineapple peel flour; (2) pineapple peel flour was steamed for 30 min to make it sterile from various kinds of microbes such as fungi and other bacteria that can interfere with the fermentation process [12]; (3) add 1% yeast culture and 10% lactic acid bacteria from the weight of pineapple peel flour; (4) a molasses solution was prepared to have a concentration of 25% (200 ml molasses: 800 ml sterile water); (5) the molasses solution was added into the pineapple peel flour that was mixed with the inoculum evenly until a whole pineapple peel flour fist was formed; (6) the pineapple peel flour and molasses were mixed in a container. The container was not tightly closed in order to create a facultatively anaerobic condition and incubated for 3–4 days at room temperature; and (7) the results of the fermentation were carried out by laboratory tests to determine the nutritional content of fermented pineapple peel.

### Fattening of male Bali cattle

The experimental cattle were placed in individual pens at random, based on a completely randomized design in one way pattern with three feeding treatments, and each treatment used four heads of male Bali cattle as replicates. The ration treatments were:

T0 = NG + (39% MC + 61% RB + 0% fermented pineapple peel).

T1 = NG + (10% MC + 70% RB + 20% fermented pineapple peel with yeast culture).

T2 = NG + (15% MC + 65% RB + 20% pineapple peel fermented with lactic acid bacteria).

The nutritional content of concentrates for each treatment is presented in Table 1.

### Meat sample preparation

The meat sample of Bali cattle used was taken from the LD muscle. The number of meat samples used was 24, representing 3 treatments and 8 replicates.

### Marbling score of male Bali beef

**Figure 1.**
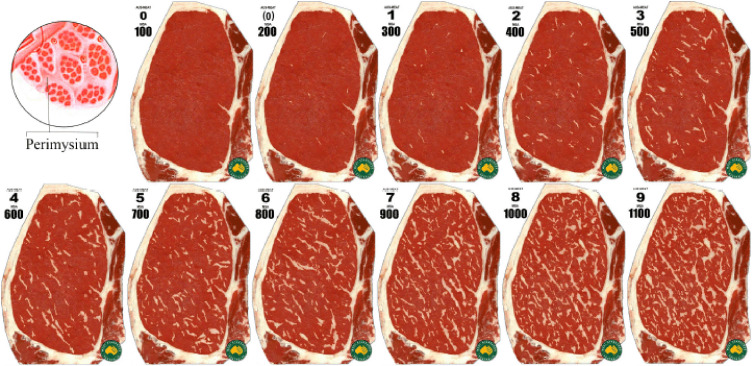
Meat marbling score standard according to AUS_MEAT and MSA [20]. AUS_MEAT = Australian meat; MSA = Meat Standards Australia. The white color between the muscle fibers is called meat marbling, while the white on each side of the muscle is called perimysium tissue. Numbers 0–9 indicate the score marbling.

**Figure 2.**
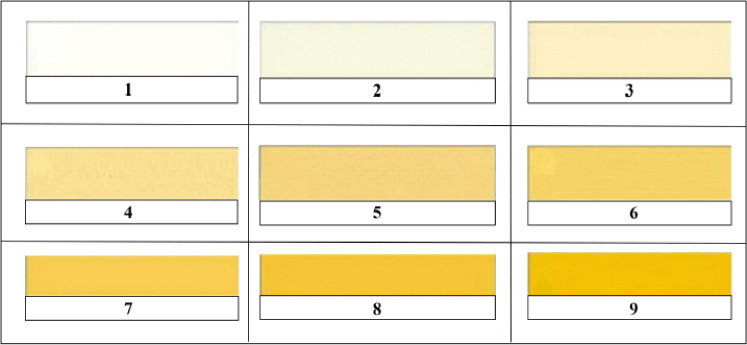
Beef fat color standard [19]. Score 1–3: white fat; score 4–6: yellowish white fat; and score 7–9: yellow fat.

**Figure 3.**
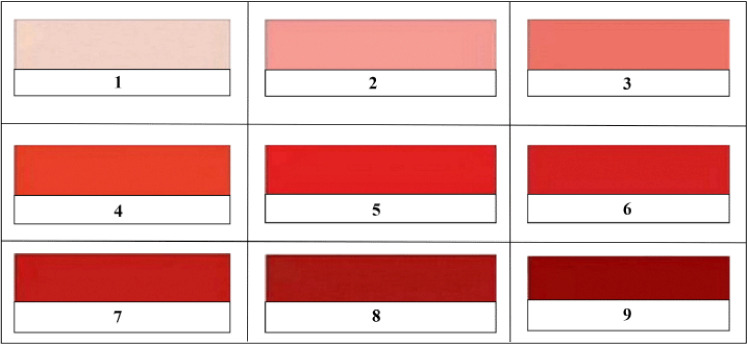
Standard of the beef color [19]. Score 1–5: bright red flesh color; score 6–7: dark red flesh color; and score 8–9: deep red flesh color.

The marbling score was measured using a comparative method based on international scores [18], while meat color and fat color were determined using the SNI standard [19]. The reference for marbling values based on global standard values is shown in Figure 1. The fat color reference is shown in Figure 2 and the meat color reference is shown in Figure 3.

The procedure for measuring marbling quality, fat color, and meat color was carried out as follows: (1) prepare meat samples of LD that consist of 50 gm of muscle per replication with a total of 24 samples for each variable; (2) meat samples were stored in the freezer for 24 h; (3) meat samples in frozen condition were cut with an area of 24 cm2; (4) observation of marbling quality, fat color, and meat color involved 20 semi-trained panelists; and (5) determination of marbling quality, fat color, and meat color is carried out by comparing the marbling conditions, fat color, and meat color of each sample with the marbling standard, fat color standard, and meat color standard (Figs. 1–3).

### Male Bali beef cholesterol profile

Testing of meat cholesterol level was carried out using the Liebermann–Buchard method [21], with the following procedure: (1) meat sample of ±0.1 gm was put into a centrifuge tube, added with 8 ml of ethanol and petroleum benzene solution in a ratio of 3:1, and stirred until the homogeneous state was achieved; (2) the meat sample solution was centrifuged for 10 min (3,000 rpm); (3) pour the supernatant into a 100 ml beaker glass and evaporate in a water bath with chloroform gradually, then pour into a graduated tube to a volume of 5 ml; (4) adding residue with 2 ml of acetic anhydride and 0.2 ml of concentrated H2SO4; (5) the residue was homogenized with a vortex and kept in the dark place for 15 min; (6) absorbance readings by spectrophotometry at a wavelength (λ) of 420 nm with a standard used of 0.4 mg/ml. The measurements of LDL and HDL were tested using the enzymatic colorimetric method [2]. The following formula was used to calculate the cholesterol levels of meat:


$$\mathrm{Cholesterol}\;\mathrm{levels}\;=\;\frac{\mathrm{Sample}\;\mathrm{Absorbance}}{\mathrm{Standard}\;\mathrm{Absorbance}}\;\times \;\frac{\mathrm{Sample}\;\mathrm{Absorbance}}{\mathrm{Standard}\;\mathrm{Absorbance}}$$

**Table 1.** The nutritional content of concentrates for each treatment

Material composition	Treatment		
	RT0^a^	RT1^a^	RT2^a^
MC (%)	39	10	15
RB (%)	61	70	65
Fermented pineapple peel with yeast culture) (%)	0	20	0
Pineapple peel fermented lactic acid bacteria (%)	0	0	20
Nutrient content of the ration^b^			
Crude protein (%)	12.01	12.00	12.09
Crude fiber (%)	4.52	6.58	7.18
Crude fat (%)	9.12	10.40	9.10
Nitrogen‐free extract (NFE) (%)	62.50	61.17	61.19
Total digestible nutrient (TDN (%)	78.76	82.20	83.46
Calcium (Ca) (%)	0.04	0.05	0.04
Phosphor (%)	0.99	1.13	1.11

a RT0 = Control ration; RT1 = First treatment ration; RT2 = Second treatment ration.

b The nutritional value of each treatment ration was calculated based on the nutritional value of the ingredients that make up the ration.

### Physical–chemical of male Bali beef

The physical properties of meat consist of WHC, cooking loss, breaking strength (tenderness), and pH value. Testing of the pH value was carried out using the Ockerman method [22], i.e., dissolving 5 gm of meat samples with 45 ml of distilled water for 2 min, and the pH meter electrode was dipped into the solution until a stable number was obtained. Water-holding capacity was measured by the Hamm method [8], i.e., a 0.3 gm meat sample was placed between two filter papers and given a weight of 35 kg for 5 min. The covered area of the meat sample and the wet area were measured with a planimeter, and the difference between the two was calculated to get the wet area (stained area − meat area). The formula for calculating the water content of meat was: MgH_2_O = [(wet area (cm^2^) × (0.0948)^−1^]−0.8. Meat water-holding capacity was calculated by the formula: Water-holding capacity = Total moisture content − [(mg H_2_O) × (sample weight)^−1^] × 100%. Meat cooking loss value can be calculated by looking at the difference between the weight of the meat before and after cooking, divided by the weight of the meat before cooking, multiplied by 100% [23]. The shear press method was used to measure breaking strength by making a rectangular meat sample with a sample cross-sectional area of 1.5 × 0.67 cm^2^ [4]. Before testing the breaking strength of the meat using a test device called a tenderometer, the meat sample was boiled for 45 min at a temperature of 70°C–750°C. The load required to break the cross-section of the meat fibers is expressed in units of kg/cm^2^. The formula for calculating the tenderness/shear force of meat is: Tenderness/Shear force = {(burden) × (1.5 × 0.67 cm^2^)^−1^} × sample weight. Testing of collagen and chemical properties of meat levels was carried out using the infrared method [24].

### Data analysis

The data were analyzed using one-way analysis of variance based on a completely randomized design with a one-way pattern [25]. Data analysis was continued with the Duncan multiple range test at a 5% confidence level using the Statistical Product and Service Solutions version 16 software program.

## Results

### Marbling score

The results of the study on the quality of marbling, fat color, and color of the meat with rations containing fermented pineapple peel are shown in Table 2. The analysis showed that fermented pineapple peel rations had a significant effect (p < 0.05) on marbling, fat color, and meat color. The following test showed that the marbling score of the meat produced by the rations containing fermented pineapple peel with lactic acid bacteria was 4.0% higher and significantly different (p < 0.05) from the marbling score at T0 (2.58%) and was not significantly higher than the marbling of male Bali beef at T1 (3.25%).

**Table 2.** Marbling score, fat color, and meat color of male Bali cattle fed fermented pineapple peel.

Variable^a^	Treatment			Significance
	T0 (Control)^b^	T1 (FCY)^b^	T2 (FBAL)^b^	
Marbling (%)	2.58 ± 0.50^A^	3.25 ± 0.16^B^	4.00 ± 0.17^B^	0.010
Meat color	2.25 ± 0.50^A^	4.25 ± 0.50^B^	4.00 ± 1.16^B^	0.011
Fat color	6.00 ± 0.00^B^	6.00 ± 0.00^B^	4.75 ± 0.50^B^	0.001

Different superscripts (A, B, C, etc.) in the same line indicate significant differences (*p* < 0.05).

a The values are the mean from eight replications.

b FCY = Fermentation by culture yeast; FBAL = Fermentation by lactic acid bacteria, T0 = control, T1 = first treatment, T2 = second treatment.

The results show that the color of the male Bali beef obtained in this study was bright red, with an average score of 2.25–4.00. Table 2 shows that the fat color of male Bali beef based on treatment ranges from 4.75–6.00. Based on the standard of meat fat color set by SNI: 3932:2008, it can be said that the color of male Bali beef fat based on the treatment is classified as yellowish white [18].

### Cholesterol profile

The study results on cholesterol, HDL, and LDL of male Bali beef with rations containing fermented pineapple peel are shown in Table 3.

The analysis showed that adding fermented pineapple peel to rations had a significant effect (*p <* 0.05) on cholesterol and HDL of male Bali beef. On the contrary, LDL had no significant effect (*p* > 0.05). The results showed that the cholesterol content of male Bali beef based on treatment ranged from 80 to 180 mg/100 gm. The following test results showed that the meat cholesterol at T0 (180 mg/100 gm) was higher and significantly different (*p <* 0.05) from T1 (133 mg/100 gm) and T2 (80 mg/100 gm).

Table 3 shows that the HDL content of male Bali beef fed rations containing fermented pineapple skin ranged from 17.67 to 70 mg/100 gm. Duncan’s test results showed that the HDL of male Bali beef at T0 (17.67 mg/100 gm) was lower and significantly different (*p <* 0.05) than at T1 (43.33 mg/100 gm) and HDL at T2 (60.00 mg/100 gm).

Table 3 shows that the average LDL levels of male Bali beef ranged from 10.00 to 15.00 mg/100 gm and were not significantly different (*p* > 0.05) between all treatments. This study’s results show that adding fermented pineapple peel with yeast culture and lactic acid bacteria solution at a level of 20% produces male Bali beef containing LDL in the normal range. This study’s results suggest that adding pineapple peel fermented by yeast culture and fermented by lactic acid bacteria solution can reduce LDL levels by 33.33% and increase HDL levels by 70.55%.

**Table 3.** Cholesterol content of the meat from male Bali cattle fed fermented pineapple peel

Variable^a^	Treatment			Significance
	T0 (Control)^b^	T1 (FCY)^b^	T2 (FBAL)^b^	
Cholesterol (mg/100 gm)	180.00 ± 10.00^B^	133.33 ± 11.55^C^	80.00 ± 20.00^A^	0.000
HDL (mg/100 gm)	17.67 ± 5.77^A^	43.33 ± 15.28^B^	60.00 ± 20.00^C^	0.032
LDL (mg/100 gm)	15 ± 10.00^A^	10 ± 6.00^A^	10 ± 6.00^A^	0.492

Different superscripts (A, B, C, etc.) in the same line indicate significant differences (*p* < 0.05).

a The values are the mean from eight replications.

b FCY = Fermentation by culture yeast; FBAL = Fermentation by lactic acid bacteria, T0 = control, T1 = first treatment, T2 = second treatment.

**Table 4.** Physical–chemical properties of the meat from male Bali Beef fed fermented pineapple peel.

Variable^a^	Treatment			Significance
	T0 (Control)^b^	T1 (FCY)^b^	T2 (FBAL)^b^	
Meat pH	5.5 ± 0.22^A^	5.6 ± 0.22^A^	5.4 ± 0.19^A^	0.334
Water-holding capacity (%)	32.15 ± 3.39^A^	34.71 ± 6.47^A^	36.10 ± 6.47^A^	0.460
Cooking loss (%)	33.93 ± 2.78^B^	31.39 ± 1.60^AB^	29.16 ± 0.73^A^	0.020
Tenderness/shear force (kg/cm2)	5.88 ± 0.16^A^	4.59 ± 0.36^A^	4.08 ± 0.19^A^	0.072
Collagen (%)	1.73 ± 0.37^A^	2.00 ± 0.61^A^	1.65 ± 0.29^A^	0.884
Crude protein (%)	22.46 ± 0.37^A^	22.51 ± 0.43^A^	22.99 ± 0.46^A^	0.152
Crude fat (%)	4.12 ± 0.82^A^	3.99 ± 0.98^A^	4.23 ± 0.036^A^	0.316
Water content (%)	72.81 ± 0.48^A^	73.01 ± 0.68^A^	72.98 ± 0.76^A^	0.931

Different superscripts (A, B, C, etc) in the same line indicate significant differences (*p* < 0.05).

a The values are the mean from eight replications.

b FCY = Fermentation by culture yeast; FBAL = Fermentation by lactic acid bacteria, T0 = control, T1 = first treatment, T2 = second treatment.

**Physical–chemical properties of male Bali beef**

The physicochemical properties of male Bali beef with fermented pineapple peel feeding are presented in Table 4. The analysis showed that the addition of fermented pineapple peel in the ration had a significant effect (*p <* 0.05) on the cooking loss of male Bali beef, while on tenderness, meat pH, water-holding capacity, water content, protein content, fat content, and meat collagen had no significant effect (*p* > 0.05). Duncan’s test results showed that the water-holding capacity of male Bali beef in treatment T2 (36.10%) was, on average, higher than the water holding capacity of beef in treatments T1 (34.71%) and T0 (32.15%). The cooking loss of male Bali beef in treatment T2 (29.16%) was smaller and significantly different (*p <* 0.05) from treatment T1 (31.39%) and T0 (33.93%). The sheer force of male Bali beef at T2 (4.08 kg/cm^2^) was lower (more tender) than at T1 (4.59 kg/cm^2^) and T0 (5.88 kg/cm^2^), but there was no significant difference (*p* > 0.05) between treatments.

Table 4 shows that adding pineapple skin from fermented lactic acid bacteria to male Bali cattle rations increased the meat’s water-holding capacity: from 32.15% to 34.71% in T0 and 36.10% in T2. The addition of fermented pineapple peel in the ration at a 20% level resulted in male Bali beef with relatively the same collagen content, namely 1.65%–2.00%, and was not significantly different (*p* > 0.05) between all treatments.

## Discussion

**Figure 4.**
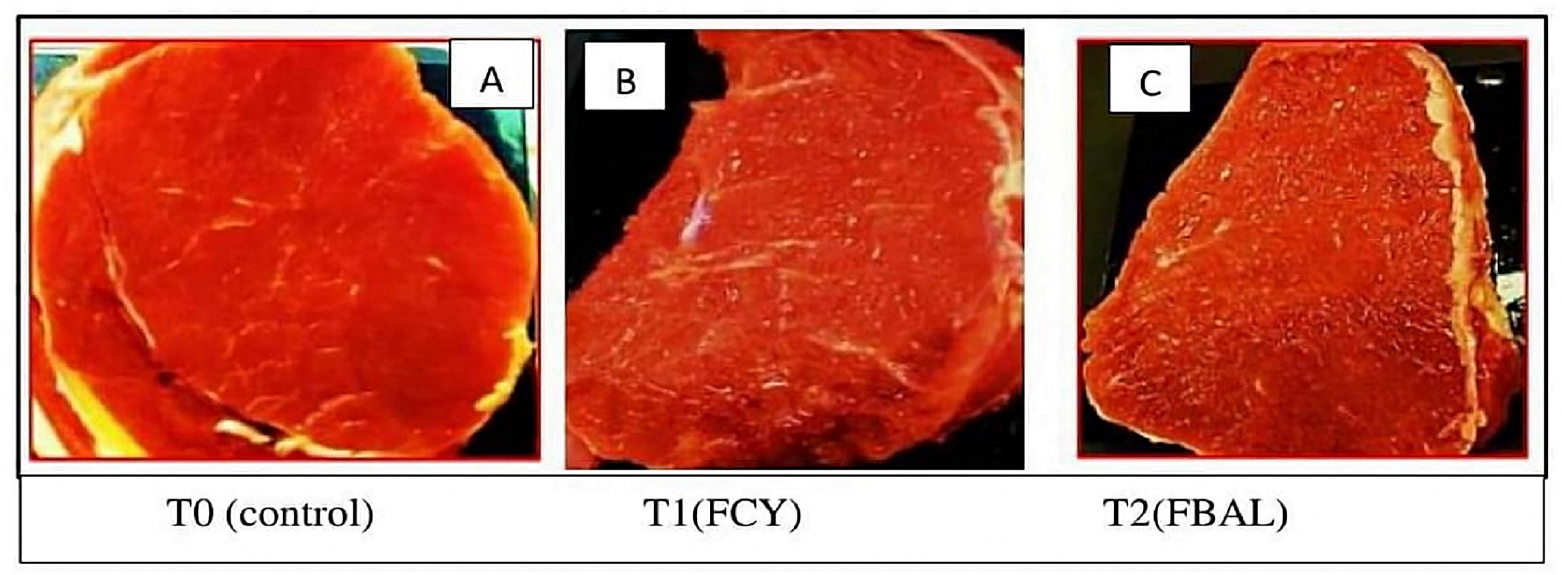
Bali beef marbling score with fermented pineapple peel feed. The meat color scores were the mean from eight replications. FCY = Fermentation by culture yeast; FBAL = Fermentation by lactic acid bacteria, T0 = control, T1 = first treatment, T2 = second treatment. A = Bali cattle meat marbling at T0 (score 2.58), B = Bali cattle meat marbling at T1 (score 3.25), and C = Bali cattle meat marbling at T2 (Score 4.00).

Based on the guidelines for assigning a meat marbling score, it was found that male Bali beef given a ration containing fermented pineapple peel ranged from 2.58 to 4.00 [18] or equal to 2.58%–4.00%, according to the US Department of Agriculture (USDA) beef grades [20]. The comparison of male Bali beef marbling scores based on treatment with the standard beef marbling score is shown in Figure 4.

Figure 4 shows that the marbling score of male Bali beef with the provision of rations containing fermented pineapple peel is 2.58%–4.00% and is relatively low (small) according to USD meat marbling standards [20]. Beef with a 2.5%–7.5% marbling score is classified as meat with a low marbling score [2]. The percentage of intramuscular fat (marbling) usually tends to increase in line with the increase in the percentage of body tissue fat, including the thickness of back fat. The nutritional status also influences the marbling content. Bali bulls fed grains will produce higher marbling than bulls fed grass [8]. Sumba Ongole cattle fed a concentrate based on bran and soybean meal can achieve a marbling score of 3 [26,27]. Male Bali cattle fed on Leucaena-based feed (a typical plant in Sumbawa) can produce meat with a marbling content of 2.4 [28].

It is very important to determine the marbling score because the marbling score can affect the tenderness and juiciness of the meat. Meat with a high marbling score causes the meat’s tenderness level to increase [29,30]. The Bali, Sumba Ongole, and Ongole Crossbred cattle in Indonesia have a marbling value ranging from 1.2 to 3.9 [31]. Nellore cattle (*Bos indicus* Brazil), Simmental cattle (Bos Taurus), Red Angus cattle, Friesian Holstein, and Caracu cattle (cattle in Brazil) have marbling scores of 3, 5.07, 5, 4.27, and 5.63, respectively [32]. Furthermore, it was reported that in Zebu cattle with Bos indicus blood, although the nature of the hot carcass dressing was high, the marbling longissimus muscle value was low. The low value of marbling in local cattle in Indonesia is caused by the large number of cattle in Indonesia originating from Bos indicus. Cattle from Bos indicus have tenderness problems related to low marbling values.

The color of the meat is one factor that determines the quality of the meat. The standard of meat color that is commonly used is the standard of beef color using a score of 1–9 ranging from pink to dark red. Score 1–5 was bright red, score 6–7 was slightly dark red, and score 8–9 was deep red [19].

The results indicate that the meat produced is bright red (score 2.25–4.0), different from the color of male Bali beef fattened with forage on smallholder farms, which has a meat color score of 9, which means deep red [33]. A male Bali cattle-fed diet based on Leucaena (*Leucaena leucocephala*) can produce bright red meat [28]. Hanwo bulls in Korea, given a concentrated 4–7 kg/day during the growth period, had good meat color with a score of 3.00 (bright red) [34]. The meat’s light color indicates that the meat’s oxygen content is still abundant; the energy reserves are still high; the slaughtering is handled according to the procedure; and the pH of the meat is still normal (5.6–5.9). Beef cattle fed moderate to high energy concentrate feed produced meat with a bright red color [33].

Critical factors that affect meat color include species, age, gender, how to cut the meat, moisture content of the meat, drying on the surface of the meat, spoilage on the surface of the meat, and light hitting the surface of the meat [35]. The bright red meat is the color of fresh beef. The bright red color of meat is due to 1) the presence of oxygen bonds to the iron atom (Fe++), which is located in the myoglobin molecular structure that dominates the surface of the meat and 2) myoglobin in meat exposed to oxygen (O_2_) reacts to form ferrousoxymyoglobin (OxyMb), causing the meat to have a bright red color [36].

Another factor that affects the color of the meat is the treatment of livestock before slaughter. Livestock that are slaughtered without resting will experience stress, causing low muscle glycogen and adenosine triphosphate (ATP) reserves. Livestock will run out of energy shortly after slaughter, and the Ca2+ content in the sarcoplasm will rapidly increase. The high content of Ca2+ causes the breakdown of muscle glycogen to last a short time so that the rigor mortis process becomes faster, resulting in a decrease in pH that is not optimal so that the final pH produced is relatively high and causes the color of the meat to become darker [37].

The color of the meat fat obtained in this study (Table 2) was white. This is because the basal diet given to the Bali cattle was fresh native grass. Male Bali cattle fed on Leucaena-based feed also resulted in meat with white color fat [28]. One factor causing beef fat’s yellowish white color is the presence of carotenoid content in native grass, especially the content of β-carotene [33]. Carotenoids are a type of pigment found in plants that is yellow, orange or reddish orange [38,39].

Animal feed and age are essential factors in determining fat color, and fat color also plays an essential role in consumer acceptance of the beef sold. For both domestic and export markets, yellow fat in carcasses is less acceptable than white fat [36]. Hanwo bulls in Korea given concentrate of 4–7 kg/day in the growth period passed fat color with a score of 4.75 (white) [34]. The fat color of the 24-month-old Aberdeen Angus bull (Hereford descendants) score is 3–5; the fat color is still relatively white [40].

High and low cholesterol levels in fresh meat are affected by several factors such as feed type, livestock, genetics, sex, and age of livestock [41]. The cholesterol levels of male Bali beef obtained in this study were considered healthy because the cholesterol levels obtained in the study were still in the range of balanced cholesterol levels and were not harmful to human health, which was less than 200 mg/100 gm [42]. The results of this study are lower when compared to the cholesterol content of male Hanwo beef in Korea, which is 248.88 mg/100 gm, with a triglyceride content of 14.63 mg/100 gm [34]. This is because the male Hanwo cattle are given feed containing concentrate at 4–7 kg/head/day.

The addition of fermented pineapple peel in male Bali cattle rations can cause the cholesterol content of male Bali beef to be lower than without the addition of fermented pineapple peel, namely in treatment T0 (180 mg/100 gm), and in treatments T1 and T2 (133.33 mg/100 gm and 80.00 mg/100 gm, respectively). This decrease in cholesterol content was due to an increase in the crude fiber content contained in the research ration; namely, the treatment T0 contains crude fiber by 4.2%, in T1 by 6.58%, and in T3 by 7.18%. The increase in the crude fiber content of the ration causes the flow rate of the ration to increase so that cholesterol in the ration will come out through bowel movements, while bile salts will be reabsorbed into the blood to be recirculated as cholesterol [12].

The pineapple peel fermentation process with yeast culture (*S. cerevisiae*) and lactic acid bacteria solution was found to be effective in reducing cholesterol levels in male Bali beef. *S. cerevisiae*, as a source of probiotics in feed, can increase the number of lactic acid bacteria, affecting the digestion process and fat absorption in the digestive tract of cattle [43]. Lactic acid bacteria in cattle’s digestive tract can utilize energy from carbohydrate sources to lower the pH of the digestive tract to 4.5 so that the digestive tract becomes acidic. The acidic environment causes the activity of the lipase enzyme to decrease, so that fat digestion is reduced and also the formation of body fat decreases.

The increase in HDL levels of male Bali beef based on treatments was still considered normal because the normal standard for blood HDL was >20 mg/dl [34] or >60 mg/dl [32]. HDL is a good cholesterol group because high HDL levels can decrease the risk of atherosclerosis by transporting cholesterol from peripheral tissues into the liver and reducing excessive cholesterol [35]. HDL is good because it has antioxidant properties that stop LDL from oxidizing [36].

Normal levels of LDL are <130 mg/dl [44] or 130–159 mg/dl [42]. Ration containing fermented pineapple peel of *S. cerevisiae* and fermented lactic acid bacteria given to male Bali cattle can be utilized optimally by releasing the enzyme bromelain to catalyze glycerol and fatty acids so that LDL is remodeled. One indicator that can be used to assess meat quality is the size of HDL and LDL values. Meat that is considered good and safe for consumption is meat that has a high HDL (good cholesterol) value and a low LDL (bad cholesterol) value [8].

The increase in water-holding capacity of meat was caused by the addition of fermented pineapple peel by lactic acid bacteria in the ration, causing the meat protein bonds called actomyosin bonds to bind to several myofibrillar protein molecules, and the amount of free water in the meat increased so that the dissolved protein was low. The results are in line with the opinion stated previously [45] that the water-holding capacity of meat is the ability of meat to hold several protein molecules and free water during external influences such as slicing, heating, grinding, or pressing. Free water has positive and negative charges that can be associated with several meat myofibrillar protein molecules, causing less free water to come out, the higher the water-holding capacity.

The water-holding capacity of male Bali beef obtained in this study ranged from 32.15 to 36.10%. The results of this study were higher than the water-holding capacity of local Sumba Ongole beef fed low to high energy feed, which ranged from 27.12% to 29.09% [26]. The high and low water holding capacity of meat depends on several factors, including the ability of muscle protein to retain free water in muscle tissue and the ultimate pH value of meat [8].

It was explained that there was a correlation between the pH value of the meat and its water-holding capacity. The increase in the pH of the meat causes the water retained in the meat’s muscle to also increase [45]. The pH of the meat obtained in this study ranged from 5.4 to 5.6, still in the normal meat pH range of 5.4–5.8, so the water-holding capacity of male Bali beef was still relatively normal. It was explained that if the pH of the meat was low, which is below 5, increased lactic acid will reduce reactive protein groups and cause more meat water to be released so that the water holding capacity of the meat decreases. Furthermore, it was said that a decrease in the pH of meat would increase actomyosin contractions and result in a reduction of water-holding capacity due to the rapid breakdown of ATP and increase the protein denaturation process. The water-holding capacity of meat will increase if the pH of meat or processed meat products has a pH range of 5.1–7 [46].

**Figure 5.**
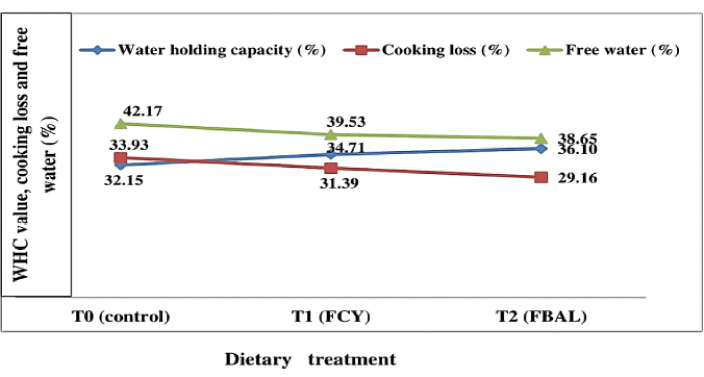
Graph of the relationship between WHC, free water, and cooking loss. The values were the replications of the mean. FCY = Fermentation by culture yeast; FBAL = Fermentation by lactic acid bacteria, T0 = control, T1 = 1st treatment, T2 = 2nd treatment, WHC = Water-holding capacity.

The average cooking loss of male Bali beef with the addition of fermented pineapple peel in the ration ranged from 29.16% to 33.91%. This study’s cooking loss of meat was in the normal range. The value of cooking loss of meat is generally between 1.5% and 54.5%, with a range of 15%–40%. The average cooking loss value of male Bali beef based on successive treatments is T0 = 33.93%, T1 = 31.39%, and T2 = 29.16%. As shown in Figure 5, there is a relationship between WHC, free water (MgH_2_O), and cooking loss of male Bali beef.

Figure 5 shows that adding fermented pineapple peel by lactic acid bacteria to the ration produces male Bali beef with the highest value of WHC (36.10%) compared to other treatments, and the lowest cooking loss value of meat was 29.16%. Figure 5 also shows that the higher the water-holding capacity of the meat, the lower the cooking loss and the free water of the meat. Meat that can hold a lot of water is considered to be of good quality.

The study shows that the meat of male Bali cattle with a low cooking loss value has better quality than male Bali beef with a high cooking loss value. Meat with a low cooking loss means that less of its nutrients will be lost when it is boiled [26].

**Figure 6.**
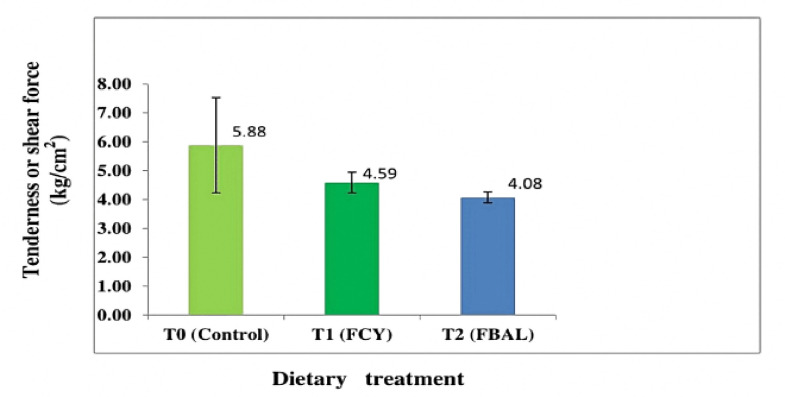
Graph of tenderness or shear force of meat (kg/cm^2^). The mean of eight replicates with standard deviation. FCY = Fermentation by culture yeast; FBAL = Fermentation by lactic acid bacteria, T0 = control, T1 = 1st treatment, T2 = 2nd treatment.

Meat tenderness is an essential factor in meat processing. One indicator to determine the level of meat tenderness can be seen from the size of the breaking power of meat fibers per unit area. The higher the breaking strength value, the more load is needed to break the meat fibers per square centimeter, which means that the meat is getting tougher or the tenderness level is lower [46]. The average tenderness/shear force of male Bali beef based on the addition of fermented pineapple peel in the ration is shown in Figure 6.

Figure 6 shows that the shear force (kg/cm^2^) required to break the bonds of meat fibers per unit area was successfully reduced from 5.88 kg/cm^2^ (T0) to 4.59 kg/cm^2^ at T1 and 4.08 kg/cm^2^ at T2. Figure 6 also explains that the bromelain enzyme contained in pineapple peel fermented with lactic acid bacteria is more effective in tenderizing meat compared to pineapple peel fermented with yeast culture. Pineapple peel is fermented by yeast culture and lactic acid bacteria after being added to the ration and eaten by male Bali cattle. This makes it easier for the nutrients in the ration to be absorbed and used to make muscle fibers or meat. This can indirectly help soften muscle fibers.The meat tenderness can be classified as very soft if the required load is less than 3.3 kg/cm^2^; soft if the load is 3.3–5.0 kg/cm^2^; slightly soft if the load is 5.0–6.7 kg/cm^2^; a bit tough if the load is 6.71–8.42 kg/cm^2^; tough if the load is 8.42–10.12 kg/cm^2^; and very tough if the load is above 10.20 kg/cm^2^ [47]. Based on the tenderness/shear force criteria, it can be said that adding fermented pineapple skin to the ration of male Bali cattle can produce tender meat.

Collagen is a connective tissue fiber that makes up the flesh along with muscle, epithelial tissue, nervous tissue, and other tissues found in muscles. In other words, collagen is a fibrous protein [8]. Collagen is the primary type of protein from the connective tissue group, where the proportion ranges from 40 to 60% [48]. It is said that collagen is a connective tissue protein that has no significant effect on the chemical composition of meat [8]. Again, it was said that collagen levels did not have much to do with the chemistry of the meat, but they did affect the amount of protein in the meat because collagen contains hydroxylysine and hydroxyproline, which are proteins [48].

Feeding fermented pineapple peel at 20% responded to the percentage of male Bali beef protein content, which was 22.46%–2.99%. This was because the nutritional content of the ratio used for all treatments was iso-protein of 12.00%–12.09%, with a total digestible nutrient of 78.76%–83.46%. The protein content of the meat, which was not significantly different, was due to the relatively similar protein consumption of the three treatments, namely 611.52–630.18 gm/head/day. Consumption of the same protein allows the fulfillment of nutrient needs for energy formation and synthesis of organic materials, including protein synthesis, that are not different, so that the treatment of fermented pineapple peel produces meat protein levels that are not different [12].

Feeding fermented pineapple peel at a level of 20% gave the same response as the percentage of the fat content of male Bali beef, which resulted in a fat content of 3.99%–4.23%. This was due to ration consumption based on the dry matter, which was not significantly different (*p* > 0.05) between treatments, which ranged from 5.09 to 5.22 kg/head/day. Consumption of relatively the same ration allows the formation of the same amount of energy. It was explained that excess energy would be converted into energy reserves in the form of glycogen and body fat, including fat in meat [49].

The water content of male Bali beef obtained in this study ranged from 72.81% to 73.01% and was still classified as a normal meat water content. It was said that normal meat has a water content of 68%–80% [9]. Male Bali ducks with the addition of papaya leaf flour in the ration at 2%, 4%, and 6% had the moisture content of the meat, respectively, of 60.92%, 60.99%, and 61.33% [50]. The water content of the meat is strongly influenced by the age of the livestock, the type of muscle, and the type of feed. It was said that the older the age of the livestock, the lower the water content and vice versa [11]. The weaknesses or shortcomings of this research are that there has been no testing on the digestibility of feed, such as protein digestibility, digestibility of organic matter, digestibility of crude fiber, and testing of the microbial content of male Bali beef by feeding fermented pineapple peel.

## Conclusion

Adding 20% of fermented pineapple peel with lactic acid bacteria to the ration can improve the quality of marbling, produce cholesterol, and the physical–chemical value of meat that meets the Indonesian National Standard.

## List of abbreviations

NG = Native grasses; MC = Milled Corn; RB = Rice Bran; LD = Longissimus Dorsi; HDL = High-Density Lipoprotein; LDL = Low-Density Lipoprotein; SNI = Indonesian National Standard.

## References

[1] Dwipayana IKB, Suryani NN, dan Mahardika IG (2019). Konsumsi nutrien, kecernaan bahan kering dan bahan organik ransum sapi bali di posko penampungan ternak desa nongan kabupaten karangasem. J Trop Anim Sci.

[2] Suryanto E, Bulkaini B, Soeparno S, Karda IW (2017). Carcass quality, marbling, meat cholesterol and non-carcass components of Bali cattle fed with fermented cacao shell. J Indonesian Trop Anim Agric.

[3] Badaruddin R, Pagala MA, Nasiu F, Hadini HA, Syamsuddin S, Akramullah M (2020). Characteristics of body weight and body size of bali cattle aged 0–8 weeks in sub-district Landono, Konawe Selatan Regency. IOP Conf. Ser.: Earth Environ Sci.

[4] Ningsi R, Asnawi A, Abdullah A (2020). Effect of Intrinsic Factors on farmers’ willingness to pay on the success of artificial insemination of Bali cattle. IOP Conf Ser Earth Environ Sci.

[5] Dahlanuddin D, Panjaitan T, Waldron S, Halliday M, Ash A, Morris S (2019). Adoption of leucaena-based feeding systems in Sumbawa, eastern Indonesia and its impact on cattle productivity and farm profitability. Trop Grassl-Forr Trop.

[6] Bahar S, Rachman R, Corfield J, Pengelly B (2020). Forage resources for Bali cattle (*Bos javanicus*) in small holder farming systems in south Sulawesi Province, Indonesia. IOP Conf Ser Earth Environ Sci.

[7] Harper K, Quigley SP, Antari R, Dahlanuddin D, Panjaitan TS, Marsetyo M (2019). Energy supplements for Leucaena. Trop Grassl-Forr Trop.

[8] Correa JE Nutritive value of goat meat. An Equal Opportunity Educator and Employer.

[9] Soeparno (2015). Ilmu dan Tenologi Daging. Edisi Revisi Cetakan ke enam.

[10] Suryanto E, Bulkaini B, Ashari A, Karda IW (2014). Carcass quality, marbling, and cholesterol content of male bali cattle fed fermented cocos shell. J Indonesian Trop Anim Agric.

[11] Bulkaini B, Wulandani W, Kisworo D, Yulianto W, Yasin M, Chotimah C (2020). The effect of slaughter age on chemical and physical characteristics of beef of Bali cattle reared extensively. Int J Pharm Res.

[12] Bulkaini B, Kisworo D, Indarsih B, Sumadi IK (2021). Production performance of peking ducks with feeding of fermented yeast culture pineapple peel (*Saccharomyces cerevisiae*). Jurnal Biologi Tropis.

[13] Bidura IGNG, Sudana IB, Mahardika IG, Suyadnya IP, Oka IGL, Aryani IAI (2012). The implementation of *Saccharomyces spp.n-2*isolate tape (isolation from traditional yeast tape) for improving feed quality and performance of male Bali duckling. Agric Sci Res J,.

[14] Masengi KIEG, Siampa JP, Tallei TE (2020). Encapsulation of the lactic acid bacteria from pineapple skin (*Ananas comous*) fermentation with staining of telang flowers (*Clitoria ternatea*). Jurrnal Bios Logos.

[15] Ariefta GA, Putra GP, dan Dewi Anggreni AA (2016). pengaruh penambahan ragi tape dan waktu fermentasi terhadap karakteristik pulpa biji kakao. Jurnal Rekayasa Dan Manajemen Agroindustri.

[16] Ibrahim W, Mutia R, Nurhayati N, Nelwida N, Berliana B (2016). Fermented pineapple peel supplementation with addition of medicinal weeds on nutrient intake consumption of broiler chicken. J Agripet.

[17] Noviandi IM, Yaman A, dan Rinidar (2017). Efek Pemanfaatan kulit nenas (*Ananas comosus*(L). Merr) dalam pakan fermentasi terhadap kandungan protein daging ayam potong. Prosiding Seminar Nasional Biotik.

[18] Yulianto W, Bulkaini B (2018). Quality of carcass, beef marbling and meat cholesterol content of male bali cattle fed with fermented cocoa pod husk-based feed. Int J Curr Adv Res.

[19] AUS-MEAT (2021). Handbook of Australian Beef Processing. Version 8 AUS-MEAT Limited.

[20] Badan Standar Nasional (2008). Mutu Karkas dan Daging Sapi.

[21] Emerson MR, Woerner DR, Belk KE, Tatum JD (2014). Effectiveness of USDA instrument based marbling measurements for categorizing beef carcasses according to differences in longissimus muscle sensory attributes. J Anim Sci.

[22] Bidura IGNG, Siti NW, Partama IBG (2019). Effect of probiotics, *Saccharomyces*spp. Kb-5 and Kb-8, in diets on growth performance and cholesterol levels in ducks. South Afr J Anim Sci.

[23] Sofiana S (2012). Penambahan tepung protein kedelai sebagai pengikat pada sosis sapi. Jurnal Ilmiah Ilmu-Ilmu Peternakan.

[24] Komansilan S (2015). Pengaruh Penggunaan Beberapa Jenis Filler Terhadap Sifat Fisik Chicken Nugget Ayam Petelur Afkir. Jurnal Zootek.

[25] AOAC (2007). Association of official analytical chemists official method. fat, moisture, and protein in meat and meat. Products.

[26] Steel RGD, Torrie JH (2017). Prinsip Dan Prosedur Statistika. Penterjemah Bambang Sumantri.

[27] Priyanto R, Fuah AM, Aditia EL, Baihaqi M, Ismail M (2015). Improving productivity and meat quality of local beef cattle through fattening on cereals based feed with different energy levels. JIPI.

[28] Khairunnisa S, Hilmia N, Novelina S, Rahmat D, Ulum MF (2019). Ultrasound imaging to estimate carcass quality of pasundan cattle based on body condition score. laporan penelitian. Fakultas Kedokteran Hewan.

[29] Tait CA, L’Abbe´ MR, Smich PM, Roe LC (2017). The association between food insecurity and incident type 2 diabetes in Canada: a population-based cohort study. PLoS One.

[30] Luo J, Sun X, Cormack BP, Boeke JD (2018). Karyotype engineering by chromosome fusion leads to reproductive isolation in yeast. Nature.

[31] Shiddieqy M, Pratiwi N, Soewandi BDP (2019). Utilization of molecular marker to improve cattle carcass quality in Indonesia.. Wartazoa.

[32] Rotta PP, Do Prado IN, Do Prado RM, Moletta JL, Silva RR, Perotto D (2009). Carcass characteristics and chemical composition of the longissimus muscle of nellore, caracu and holstein-friesian bulls finished in a feedlot. Asian-Austral J Anim Sci.

[33] Tahuk PK, Dethan AA, Sio S (2020). Meat and fat colors characteristics of male bali cattle fattened with green feed in smallholder farms.. J Trop Anim Sci Technol.

[34] Chung CS, Cho WK, Jang IS, Lee SS, Moon YH (2017). Effects of feeding system on growth performance, plasma biochemical components and hormones, and carcass characteristics in hanwoo steers. Asian-Austral J Anim Sci.

[35] Purdue University of Sciences (2012). http://ag.ansc.Purdue.edu/meat_quality/marbling_consumer.html.

[36] Kuntoro B, Maheswari RRA, Nurain H (2013). Mutu fisik dan mikrobiologi daging sapi asal rumah potong hewan (RPH) kota pekanbaru. J Peter.

[37] Bintoro Nurwantoro VP, Legowo AM, Purnomoadi A (2012). Pengaruh metode pemberian pakan terhadap kualitas spesifik daging. Review. Jurnal Aplikasi Teknologi Pangan.

[38] Pursetyo KT (2020). Analisis Klorofil dan Karotenoid Pada Alga.

[39] Hughes JM, Kearney G, Warner RD (2014). Improving beef meat colour scores at carcass grading. Anim Prod Sci.

[40] Lisitsyn AB, Kozyrev K (2016). Researching of meat and fat colour and marbling in beef.theory and practice of meat processing.

[41] Gokce MA, Tazbozan O, Celik M, Tabakoglu S (2004). Seasonal variation in proximate and fatty acid of female common sole (*Solea solea*). Food Chem.

[42] Tugiyanti E, Heriyanto S, Syamsi1 AN (2016). Effect of soursop (*Announa muricata**L*) leaf meal on characteristics of blood and meat fat of native male tegal duck. Bul Peter.

[43] Bidura IGNG, Candrawati DPMA, Warmadewi DA (2015). Selection of khamir *Saccharomyces*spp. isolated from colon of native chickens as a probiotics properties and has CMC-ase activity. J Biol Chem Res.

[44] Basmacioglu H, Ergul M (2005). Research on The factor affecting cholesterol content and some other characteristics of eggs in laying hens. Turk J Vet Anim Sci.

[45] Cahyanti AN, dan Rohadi Iswoyo (2020). Perubahan Daya Ikat Air, Tekstur, pH, dan total mikroba pada daging ayam segar yang direndam dengan larutan ekstrak kunyit. prosiding seminar teknologi dan agribisnis peternakan vii–webinar; prospek peternakan di era normal baru pasca pandemi COVID-19. Fakultas Peternakan Universitas Jenderal Soedirmanm.

[46] Bulkaini B, Kisworo D, dan Yasin M (2019). Physical characteristics and organoleptic values of horse’s meat sausage based on level subtitution of tapioca flour. J Vet.

[47] Komariah K, Rahayu S, Sarjito S (2019). Physical characteristics of beef, buffalo and lamb meat on different postmortem periods. Bul Peter.

[48] Feiner G (2006). Meat products handbook: practical science and technology. Woodhead Publishing Limited, Cambridge, UK,.

[49] Kartikasari LR, Nuhriawangsa AMP, Ratriyanto A (2003). Chemical composition of duck meat refused with different frequency feeding. Bul Peter.

[50] Siti NW (2016). Meningkatkan kualitas daging itik dengan daun pepaya, cetakan pertama. diterbitkan oleh swasta nulus bekerjasama dengan bali shanti pusat pelayanan konsultasi adat dan budaya Bali (LPPM UNUD).

